# Spectral Filter Selection Based on Human Color Vision for Spectral Reflectance Recovery

**DOI:** 10.3390/s23115225

**Published:** 2023-05-31

**Authors:** Shijun Niu, Guangyuan Wu, Xiaozhou Li

**Affiliations:** 1 Faculty of Light Industry, Qilu University of Technology (Shandong Academy of Sciences), Jinan 250353, China; 10431210990@stu.qlu.edu.cn; 2 State Key Laboratory of Biobased Material and Green Papermaking, Qilu University of Technology (Shandong Academy of Sciences), Jinan 250353, China; xzl@qlu.edu.cn

**Keywords:** multispectral acquisition system, filter selection, spectral recovery, human color vision, weighted area selection, custom error score ranking

## Abstract

Spectral filters are an important part of a multispectral acquisition system, and the selection of suitable filters can improve the spectral recovery accuracy. In this paper, we propose an efficient human color vision-based method to recover spectral reflectance by the optimal filter selection. The original sensitivity curves of the filters are weighted using the LMS cone response function. The area enclosed by the weighted filter spectral sensitivity curves and the coordinate axis is calculated. The area is subtracted before weighting, and the three filters with the smallest reduction in the weighted area are used as the initial filters. The initial filters selected in this way are closest to the sensitivity function of the human visual system. After the three initial filters are combined with the remaining filters one by one, the filter sets are substituted into the spectral recovery model. The best filter sets under L-weighting, M-weighting, and S-weighting are selected according to the custom error score ranking. Finally, the optimal filter set is selected from the three optimal filter sets according to the custom error score ranking. The experimental results demonstrate that the proposed method outperforms existing methods in spectral and colorimetric accuracy, which also has good stability and robustness. This work will be useful for optimizing the spectral sensitivity of a multispectral acquisition system.

## 1. Introduction

Over the last few decades, multispectral imaging technology has been widely used because it solves the “metameric issues” problem of a traditional three-color digital imaging camera and realizes the real recording of spectral information on the surface of objects. This technology has been gradually applied in museums, art galleries, computer graphics, spectral detection, etc. [1,2,3,4,5,6]. One of the most important components in a multispectral acquisition system is the set of optical filters that allows for acquisition in different bands of the visible light spectrum. The selection of a specific filter set from a given filter space clearly affects the accuracy of spectral recovery. Although using more filters usually improves the accuracy of the spectral recovery, it also increases the operational complexity, image acquisition time, and data volume accordingly. Therefore, some scholars have conducted a significant amount of research on how to achieve the optimal selection of filters.

Filter set optimization has already been studied in some cases, but there are still many problems to be solved [7,8,9,10,11]. Some scholars have designed filters with optimal spectral sensitivity in theory based on specific optimization criteria [12,13,14,15]. However, the comprehensive effect of the optical path, light source, and sensor spectral characteristics makes the design process more complex. At the same time, the actual filter does not guarantee that the theoretically designed optimal filter has exactly the same spectral sensitivity. Another option is to select the best filter from the available filters. This exhaustive method is practical when the total number of filters are small. However, with the increase of the total number of filters to be selected, the computational complexity of the exhaustive method increases dramatically, which makes it inapplicable [16].

With extensive research, multispectral filters no longer simply rely on empirical methods to select filter compositions. The filter vector analysis method (FVAM) [17] is a commonly used method for filter selection. Hardeberg first used the maximum linear independence (MLI) [18] method to select the spectral training set, and since then Li has applied it to filter selection [19]. The selection principle of the MLI method is that the transmission matrix of the selected filter set has the smallest number of conditions. The transmission vector maximization orthogonal method (MaxOr) [20] involves selecting the filter with the largest transmission vector norm as the preferred filter, and then using each filter to form the transmission space and select the filter set with the largest transmission space orthogonality. The linear distance maximization method (LDMM) [21] uses the linear distance between filter sensitivity vectors as the only criterion for selecting filter sets. FVAM directly selects filters to form filter sets by the mathematical properties of filter sensitivity curves, which is simple and time-saving, and the stability of the selecting results is better than that of the empirical method. 

However, the above method does not consider other parameters in a multispectral imaging system, such as the spectral distribution of the light source (SPD), the spectral sensitivity of the camera, and the characteristics of the imaging scene [16,22]. This leads to the fact that although the filter chosen by FVAM can guarantee the effectiveness of the first channel response of the multispectral camera, it is difficult to satisfy the optimization requirements of the whole system. Therefore, it is necessary to develop an optimized filtering method that integrates other factors in a multispectral imaging system and selects filter sets based on the spectral recovery effect and colorimetric accuracy of each group as a reference. 

In response to the problems of the above study, a filter selection method combining weighted area selection and custom error score ranking is proposed in this paper. The method can be divided into two parts:

1. The original sensitivity curve of the filter is weighted by the LMS cone response function. The area reduction rate of the filter before and after weighting is calculated, and the minimum area reduction rate is selected. The initial filters selected in this way are closest to the sensitivity function of the human visual system.
2. The three initial filters are combined with the remaining filters one by one, and each combination is substituted into the spectral reconstruction model to obtain the recovery results of the whole imaging system. The respective optimal filter sets under L-weighting, M-weighting, and S-weighting are selected according to the customized minimum recovery error, and then the optimal filter set is selected from the three optimal filter sets by comparing the error set scores.

The innovation of the paper is to use the human visual system weighting in the filter vector analysis process so that the selected filters are closest to the human eye sensitivity curve. Other factors are integrated into the multispectral imaging system, and the filter sets are selected according to the spectral recovery effect and chromatic accuracy of each group.

## 2. Materials and Methods

Spectral recovery needs to simulate the camera response process. This spectral imaging model is suitable for any known camera response process with spectral sensitivity. The multispectral imaging model can be described by Formula (1):

(1) $$P_{i}=\int_{400}^{700}E\left(\lambda \right)R_{j}\left(\lambda \right)Q_{i}\left(\lambda \right){d\lambda +N}_{i}\;,$$

where $P_{i}$ is the response value of the *i*th channel of the sensor, $E\left(\lambda \right)$ is the illumination spectral power distribution (SPD) for each wavelength, $R_{j}\left(\lambda \right)$ is the spectral reflectance of sample *j*, $Q_{i}\left(\lambda \right)$ is the spectral sensitivity of the *i*th channel of the sensor, *λ* is the wavelength, and the sampling range is 400–700 nm, *N_i_* is the noise of the digital camera. According to Liang’s study [23], in order to simplify the calculation, the imaging model used in this paper does not consider the noise of the camera and assume that the illumination are equal power distribution. Formula (1) can be expressed in matrix form:

(2) P=M⋅R ,

where *P* is the responses matrix, *M* is the overall spectral sensitivity function matrix of the multispectral imaging system including the product of the matrix form of $E\left(\lambda \right)$, $R_{j}\left(\lambda \right)$ and $Q_{i}\left(\lambda \right)$, and *R* is the original spectral reflectance matrix.

Spectral recovery is a process of obtaining high-dimensional spectral reflectance with low-dimensional response values. There are various spectral recovery methods, such as the most common pseudo-inverse methods [24,25], principal component analysis methods [26], compressive sensing [27,28], Wiener estimation methods [29], and other methods [30,31,32,33,34]. This method used in this paper is the pseudo-inverse method, which can be expressed by Formula (3):

(3) $$R={\left(M^{T}\right)}^{-1}\cdot P$$

where ‘*T*’ is the transposition of the matrix, and ‘−1’ represents the inverse operation of the matrix. The pseudo-inverse method is used for spectral recovery. Firstly, the transformation matrix is calculated by training samples, and then the spectral reflectance of the testing sample is recovered by the transformation matrix of the known camera response.

## 3. The Proposed Method

**Figure 1:**
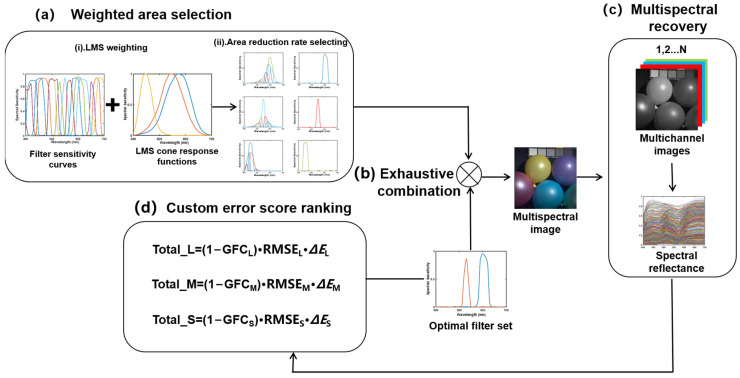
Filter selection operation schematic chart.

In this section, the flow chart of the proposed filter selection method is shown in Figure 1. The root mean square error (RMSE), goodness of fit coefficient (GFC), and color difference (Δ*E*) are used to evaluate the spectral recovery effect of the selected filter set.

This selection method can be divided into four main processes: weighted area selection, exhaustive combination, multispectral recovery, and custom error score ranking. 

Figure 1a of the flowchart is the first step of the operational process, where the original filters are weighted using the LMS cone response function. The three filters that select the best match to the LMS cone response function of the human vision system are selected as the initial filters, which are based on the morphological and mathematical characteristics of the spectral sensitivity curves of the weighted filters. This process requires the calculation of the filter area reduction rate and the selection of the initial filters based on the area reduction rate. 

Figure 1b of the flowchart shows the second step of the operational process, in which the remaining filters are exhaustively combined with the initial filters one by one after the initial filters are identified. This step is an iterative process. When selecting the filters for the next channel, the L-weighted filter set, the M-weighted filter set, and the S-weighted filter set that performed best in the previous selecting process are used as the initial filter set, respectively. The remaining filters are combined with the initial filter set one by one in an exhaustive manner. 

Figure 1c of the flowchart is the third step of the operation flow. The third step is to recover the spectra of each group of filter combinations generated by the exhaustive method in the second step one by one, and to derive the recovery error and chromaticity error of each group of filters. 

Figure 1d of the flowchart is the first step of the operational process, and the optimal filter sets under L-weighted, M-weighted and S-weighted are selected according to the custom recovery error ranking. The optimal filter sets from the three optimal filter sets are selected by comparing the custom error score ranking. When the number of channels increases, the respective optimal filter sets under L-weighted, M-weighted and S-weighted are exhaustively combined with the remaining filters, and the second step is repeated according to the number of channels until the number of filters in the selected filter set equals the number of channels. 

### 3.1. Weighted Area Selection

There are three distinct photoreceptor cells on the retina of the human eye. The three optic cones are called *L*, *M*, and *S* cones because they roughly correspond to the long, medium, and short wavelength range of the visible spectrum. In this paper, the filter is weighted by *L*, *M*, and *S* to better match the selected filter set with the human visual. The weighting process is as shown in Formula (4):

(4) $$B_{n}=V_{n}b_{i},$$

where *b* denotes the original sensitivity curve of the filter; *i* denotes the *i*th filter; *V* denotes the cone response function; *n* denotes the number of cone response functions, and *B* denotes the filter sensitivity function weighted by the LMS cone response functions.

Formula (4) uses the response value of the LMS cone response functions to weight the filter at the same time, because of the sensitivity of the human eye in different wavebands, so this paper will consider using the waveband distance between the filter peak and the LMS curve peak to weight the filter, and the process is shown in Formulas (5) and (6).

(5) $$c_{n}=\left|b_{\max}{}_{i}-V_{\max}{}_{n}\right|$$

(6) $$C_{n}=c_{n}b_{i},$$

where $b_{max}$ denotes the band of the wave peak of the filter response value; *V*_max_ denotes the band of the wave peak of the LMS cone response functions; $c_{n}$ denotes the distance between the filter peak and the LMS curve peak of the waveband, and *C_n_* denotes the filter sensitivity curve weighted by the band distance. The two-weighted filter sensitivity function is combined to obtain the final weighted filter sensitivity curve *D_n_*, such as in Formula (7).

(7) $$D_{n}=B_{n}C_{n}$$

In this step of the operation, with the spectral band for the horizontal axis, the filter sensitivity response value for the vertical axis calculates the area of the filter sensitivity curve before and after weighting, then subtracts the original area from the weighted area to get the area difference. Dividing the area difference from the original area, the area decreases the smallest filter, which is the selection of the preferred filter. The selecting process is as described in Formula (8).

(8) $$Z_{n}=\arg m\underset{n}{i}n\left(\frac{S_{i}-S_{w_{i}}}{S_{i}}\right),$$

where *S* represents the area of the unweighted filter; and $S_{w}$ represents the area of the weighted filter. By using Formula (8), we can obtain three preferred filters that best match the human eye’s *L*, *M* and *S* cone cells.

### 3.2. Exhaustive Combination

We define *b*_1_, *b*_2_ and *b*_3_ as the three preferred filters that best match the human eye after weighting the *L*-cone response function, *M*-cone response function, and the *S*-cone response function, respectively. The remaining filter is combined with the three filters separately, as shown in Formula (9).

(9) $$K_{1}={\left[b_{1},b_{i}\right]}_{}\;(i\neq 1)\;K_{2}={\left[b_{2},b_{i}\right]}_{}\;(i\neq 2)\;K_{3}={\left[b_{3},b_{i}\right]}_{}\;(i\neq 3),$$

where *K*_1_*, K*_2,_ and *K*_3_ respectively denotes the remaining filter set consisting of *b*_1_, *b*_2,_ and *b*_3_. When selecting the filters for the next channel, the initial filter set selects the best performance to the previous selecting process in the *L*-weighted filter set, *M*-weighted filter set, and *S*-weighted filter set, respectively. The remaining filters are then combined with the initial filter set one at a time in an exhaustive manner.

### 3.3. Multispectral Recovery

After obtaining the filter set, the similarity between the training samples and the testing samples in the experiment also affects the final recovery accuracy. Therefore, the Euclidean distance between the response value of the training samples and the testing samples is used as a weighting function to optimize the recovery process and express it by using Formula (10).

(10) $$s_{j}=\sqrt{{\left(p_{1}{}_{test}-p_{1}{}_{train,\;j}\right)}^{2}+{\left(p_{2}{}_{test}-p_{2}{}_{train,\;j}\right)}^{2}+\cdots +{\left(p_{i}{}_{test}-p_{i}{}_{train,\;j}\right)}^{2}},$$

where the $p_{test}$ is the response of the testing sample; the $p_{train}$ is the response of the optimal local training sample; the subscript *j* is the *j*th sample of the training sample; and the $s_{j}$ represents the Euclidean distance between the *j*th training sample and the testing sample. The order is then ascending according to the distance between the training and testing samples. The first *N* (1 ≤ *N* ≤ *j*) training samples are selected as the local optimal training samples, and the inverse distance weighting (IDW) coefficient $w_{k}$ is calculated for each selected local optimal training sample, as shown in Formula (11).

(11) $$w_{k}=\frac{1}{s_{k}+\varepsilon },$$

where the subscript *k* is the *k*th sample of the local optimal training sample; $s_{k}$ is the Euclidean distance between the *k*th local optimal training sample, and the testing sample; ε is a very small added value to avoid dividing the equality by zero, and ε = 0.001 is used. The weighted matrix *W* is defined as in Formula (12).

(12) $$W={\left[\begin{array}{cccc} w_{1} & 0 & \cdots & 0\\ 0 & w_{2} & 0 & 0\\ \vdots & 0 & \ddots & \vdots \\ 0 & 0 & \cdots & w_{k} \end{array}\right]}_{k\times k}$$

The transpose matrix *M* in the spectral recovery Formula (2) can be expressed as:

(13) $$M=R_{Train}W{\left(P_{Train}W\right)}^{-1}$$

(14) $$R={MP}_{Test},$$

where superscript ‘−1’ represents the matrix violation; *R_Train_* is the optimal spectral reflectance of the selected local training sample; *P_Train_* is the normalized response value of the training sample; *P_Test_* represents the normalized response value of the test sample; and *R* is the corresponding reconstructed spectrum.

### 3.4. Custom Error Score Ranking

Through the spatial vector analysis (FVAM) of the weighted filter, and only considering the characteristics of the filter itself, the three preferred filters have the best match for the visual sensitivity function of the human eye. The spectral recovery error and colorimetric error are calculated for each filter combination by combining the filter with other influence parameters in the multispectral acquisition system, and a custom minimum recovery error is used to select the optimal filter set.

The recovered spectra of the filter sets are obtained in Formula (3). The root mean square error (RMSE), goodness of fit coefficient (GFC), color difference (Δ*E*), peak signal-to-noise ratio (PSNR), and spectral angle map (SAM) are calculated and normalized. The recovery error is calculated as shown in Formula (15).

(15) $$TOTALn_{i}=RMSE_{i}\times (1-GFC_{i})\times \Delta E_{i}\times PSNR_{i}\times SAM_{i},$$

where *TOTALn_i_* is the custom recovery error corresponding to the *i*th filter set consisting of the *n*th preferred filter. The *RMSE*, *GFC*, Δ*E*, *PSNR* and *SAM* is calculated by Formulas (16)–(20).

(16) $$RMSE=\sqrt{\frac{1}{m}(R_{test}-R{)}^{T}(R_{test}-R)}$$

(17) $$GFC=\frac{R_{test}{}^{T}R}{\left\|R_{test}{{}^{T}}_{}R_{test}\right\|\cdot \left\|\mathrm{RR}\right\|}$$

(18) $$\Delta E^{*}{}_{ab}=\sqrt{{\left(\Delta L^{*}\right)}^{2}+{\left(\Delta a^{*}\right)}^{2}+{\left(\Delta b^{*}\right)}^{2}}$$

(19) $$PSNR=20_{}\;{log}_{10}\left(\frac{1}{RMSE}\right)$$

(20) $$SAM=\cos^{-1}(GFC)$$

As shown in Formula (21), the filter set with the smallest custom recovery error is selected as the optimal filter set under the current number of channels.

(21) $$G_{n}=argm\underset{n}{i}n\left({TOTALn}_{i}\right),$$

where *G_n_* represents the optimal filter set under the current number of channels. The Formulas (9) and (15)–(21) process can be repeated according to the number of channels in the multispectral imaging system.

## 4. Experiment

To evaluate the performance of the method, comparative experiments are performed based on both simulated and actual multispectral acquisition systems. Four metrics are used to assess the accuracy of the recovery. CIE DE1976 (∆E*_ab_) and CIE DE2000 (∆E*_00_) are used as the reference indices to measure color difference. The root mean square error (RMSE) and goodness of fit coefficient (GFC) are used as the spectral reflectance indices. 

### 4.1. Simulation Experiment

**Figure 2:**
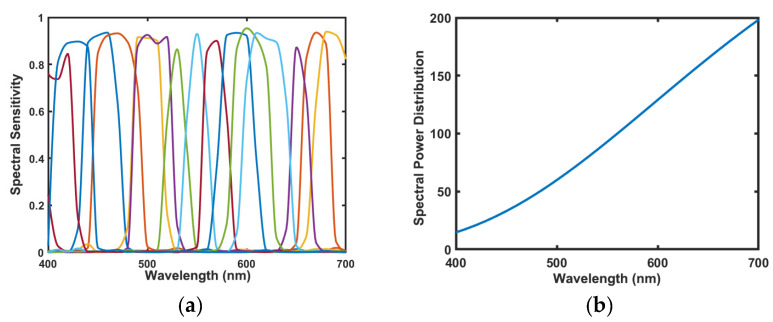
(**a**) Filter spectral sensitivity; (**b**) the spectral power distribution of CIE illuminant A.

To verify the performance of the proposed method, simulation experiments are first performed using a simulated multispectral acquisition system. The systematic noise treatment is not considered in the simulation experiments [23]. The filter data set comes from 15 filters designed at equal intervals by our laboratory. The sensitivity vectors are shown in Figure 2a. We used the CIE illuminant A as the reference light source, while the spectral power distribution of the light source is shown in Figure 2b. Each curve in Figure 2a represents the spectral sensitivity of a filter, and the different color curves represent different filters.

The 1269 of Munsell Matt chips [35], 140 Color Checker SG [36], and the 354 Vrhel spectral dataset [37] are used in a simulation experiment. In order to make the experimental results more convincing, Munsell Matt chips are used as the training sample. The Munsell Matt chips, Color Checker SG, and the Vrhel spectral dataset are used as the testing samples. The spectral reflectance ranges from 400 to 700 at 10 nm intervals.

**Figure 3:**
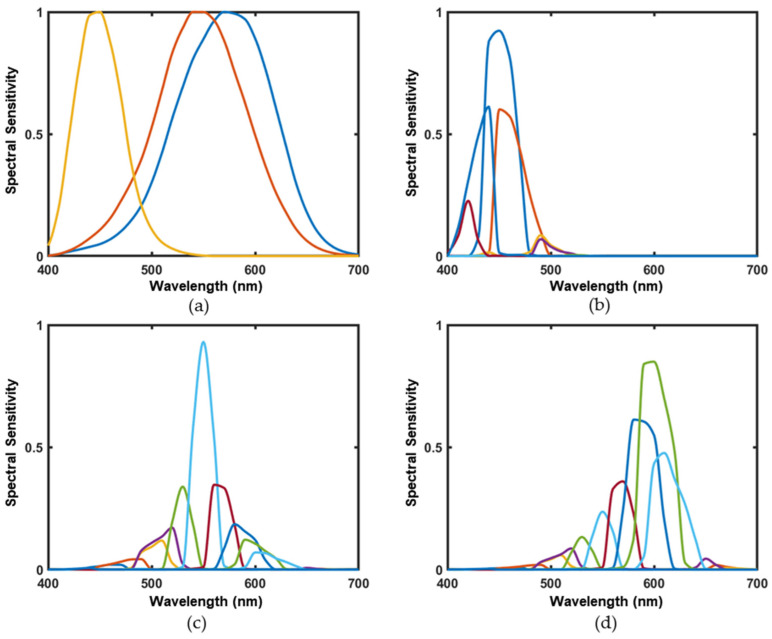
(**a**) the LMS cone response functions; (**b**) the filter transmission weighted by the *S* response curve; (**c**) the filter transmission weighted by the *M* response curve; and (**d**) the filter transmission weighted by the *L* response curve.

Before selecting the preferred filter, the LMS cone response functions are shown in Figure 3a, and the filter curve weighted by LMS cone response function is shown in Figure 3b–d. The different color curves represent different filters.

After the three preferred filters are obtained by the weighted area selection, the remaining filters are combined with the preferred filter to form a filter set. The spectral recovery error and colorimetric error are calculated and multiplied by each error parameter index, from which the filter set with the smallest custom error value is selected as the optimal filter set. Therefore, after the analysis of the spectral information data, the response value of the data information should also be analyzed.

**Figure 4:**
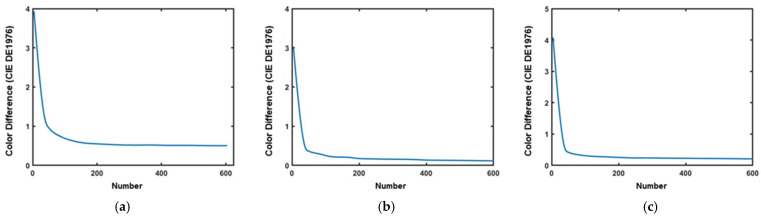
(**a**) the relationship between CIE DE1976 color difference and the number of local optimal training samples in Munsell Matt chips; (**b**) the relationship between CIE DE1976 color difference and the number of Color Checker SG training samples; and (**c**) the relationship between CIE DE1976 color difference and the number of local optimal training samples in Vrhel spectral dataset.

Before verifying the parameter indicators of the final selected filter set, the samples first need to be optimized first, and then an appropriate number of characteristic samples should be selected for spectral recovery according to the distance between the samples. To obtain the optimal parameters, the effect of contrast color error in the number of locally optimal training samples is explored, and the results are shown in Figure 4. According to Figure 4, 150 is selected as the number of locally optimal training samples in this experiment.

This study compares the spectral recovery accuracy and colorimetric accuracy of this method with three other existing methods under the same experimental conditions, and the results are shown in Table 1. We compare the recovery results of three samples under the same light source, thus verifying the performance of the method under different shooting conditions. The experimental conditions in Table 1 result from using the Munsell Matt chips as the training samples and the testing samples, selecting the 3–7 channels, and using the other methods under the CIE illuminant A.

The experimental results in Table 1 show that the maximum and mean color difference are the smallest under the different number of channels, and the proposed method is superior to other methods in terms of colorimetric metrics. In terms of spectral recovery, both the RMSE and GFC evaluation indices of the proposed method are better than the existing methods, which also means that this method has a good spectral recovery effect.

**Figure 5:**
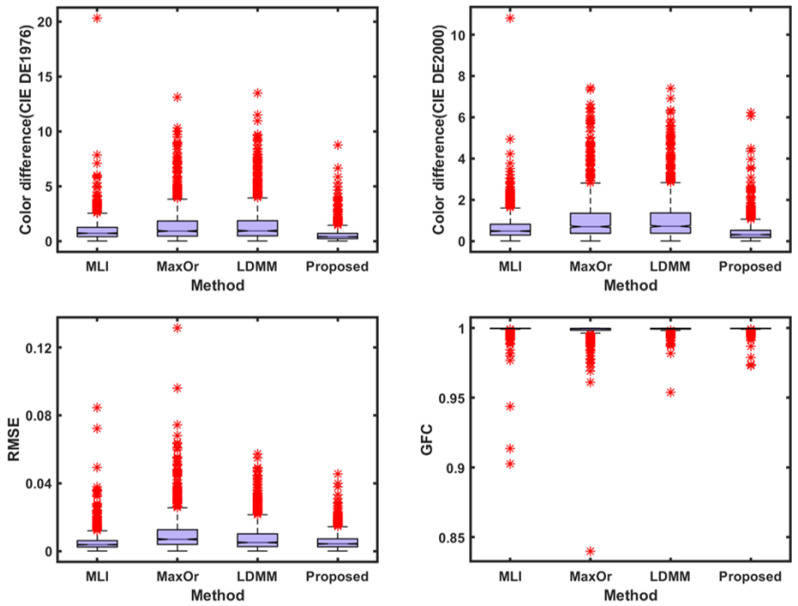
A box plot of each parameter index of Munsell Matt chips under CIE illuminant A.

To make the results more intuitive and to visualize the recovery data, this study used box plots to demonstrate the spectral and colorimetric recovery accuracy under the different methods, as shown in Figure 5. The boxplot is a standardized way of displaying the spectral recovery results, which are the minimum, maximum, median, and first and third quartiles. The value closest to the box indicates the best spectral recovery results, while the value farther from the box indicates the worst spectral recovery results. The * in the figure represents the anomaly, the farther the anomaly is from the box, the worse the spectral reconstruction effect. The box of the boxplot of the proposed method is smaller than other methods, and shows the best results in the maximum and mean. This more intuitively shows that the proposed method is superior to other methods.

**Figure 6:**
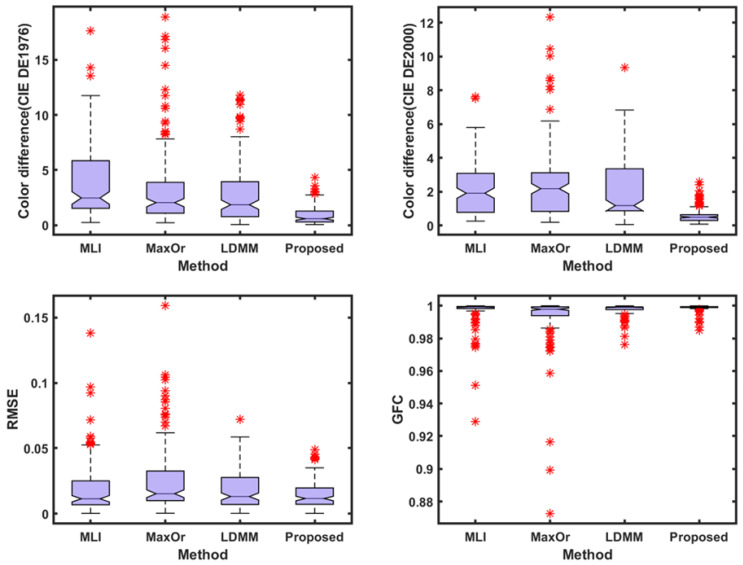
A box plot of each parameter index of Color Checker SG under CIE illuminant A.

The experimental conditions in Table 2 are the result of using the Munsell Matt chips as the training sample at the CIE illuminant A, using the Color Checker SG as the testing sample, and selecting the 3–7 channels and the other methods used here. The box plots are shown in Figure 6.

**Figure 7:**
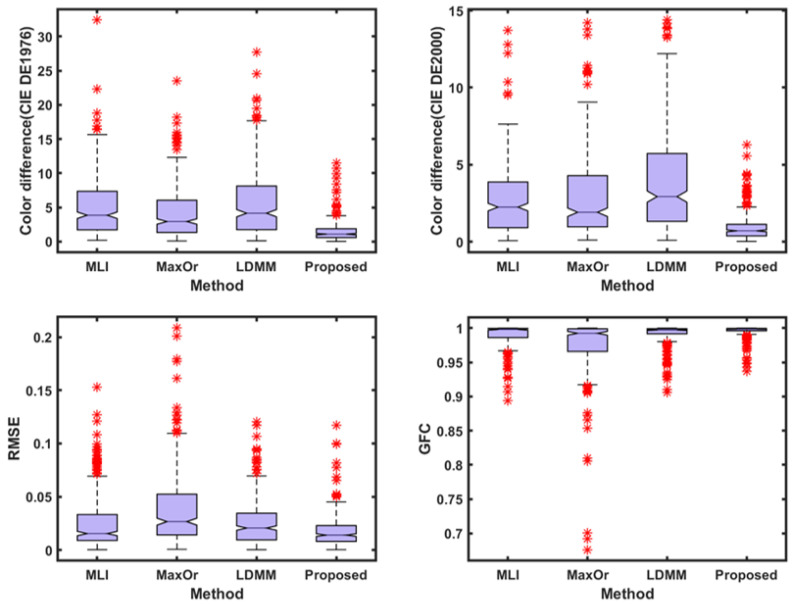
A box plot of each parameter index of the Vrhel spectral dataset under CIE illuminant A.

With regard to Table 2 and Table 3, and Figure 6 and Figure 7, compared with the results in Table 1 and Figure 5, the spectral recovery accuracy and colorimetric accuracy are consistent with the Munsell Matt chips, and the proposed method still outperformed the other methods. This indicates that the presented method performs better and is more stable under different samples.

**Figure 8:**
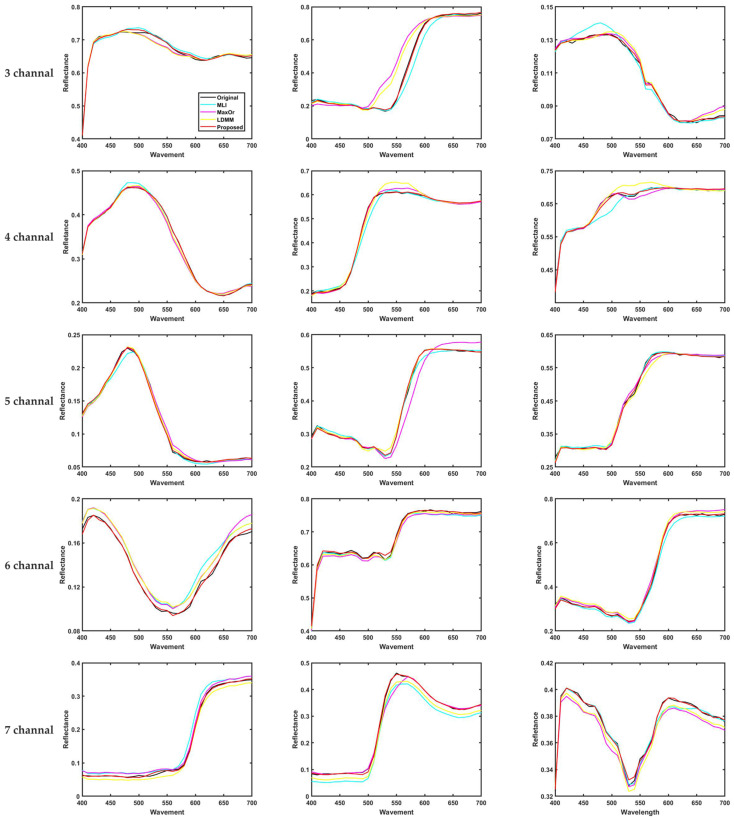
Spectral reflectance recovery results from our proposed and existing methods with three randomly selected samples.

In Figure 8, we randomly selected three samples of Munsell Matt chip training samples under CIE illumination A after spectral recovery using different methods in order to compare the results of spectral reflectance curve recovery at 3-7 channels. It can be seen that the method is closer to the original sample and has better performance.

After simple verification of the proposed method, and in order to show its good performance, it was applied to the spectral images [38].

**Figure 9:**
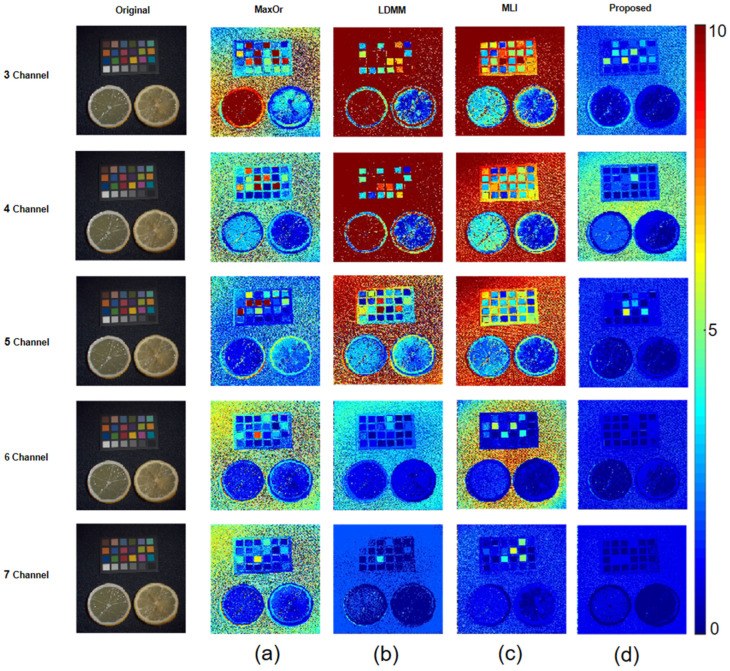
A results comparison of spectral images of different methods using the CIE 1964 color matching function as the spectral sensitivity; (**a**) MaxOr; (**b**) LDMM; (**c**) MLI; and (**d**) Proposed.

**Figure 10:**
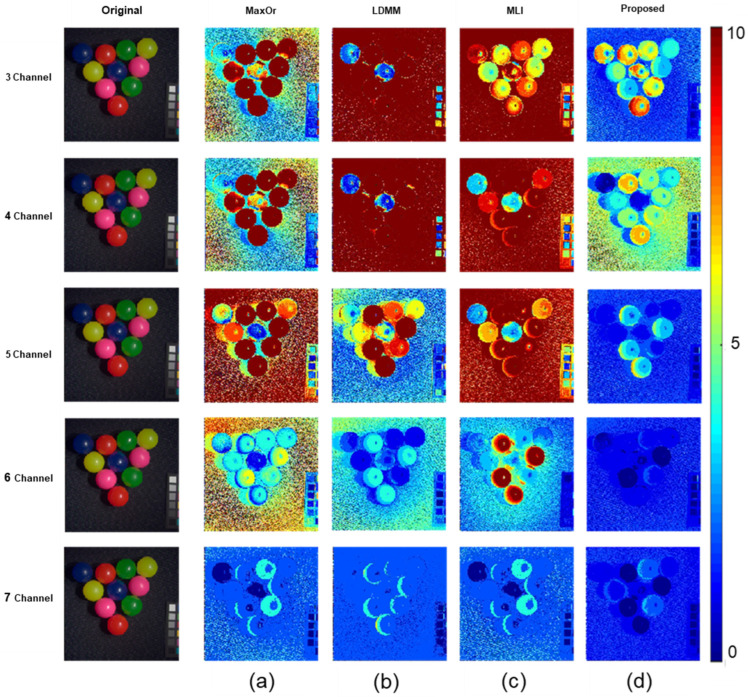
Results comparison of spectral images of different methods using the CIE 1964 color matching function as the spectral sensitivity (**a**) MaxOr; (**b**) LDMM; (**c**) MLI; and (**d**) Proposed.

Figure 9 and Figure 10 depict two images selected from the CAVE Multispectral Image Database. The first multispectral image comes from the library in the database. The image content in this library is a common object in daily life. The second multispectral image comes from the real and fake library in the database. The image content in this library is obtained by putting real objects and imitations in life together. These two pictures are of common scenes from daily life. 

It can easily be seen in Figure 10 that the results comparison of the spectral images uses the different methods to recover the spectral reflectance. Figure 9a represents the original RGB image. Figure 9 b–f is called the error map, which calculates the color difference of the spectral reflectance recovered by different methods. More red means a larger color difference, and more blue means a lesser color difference. A side-by-side comparison shows the effect of different methods for spectral image recovery, and by the color change, it can be seen that the method approach proposed in this paper is superior to other methods. Therefore, the proposed method shows better performance.

### 4.2. Real Experiment

**Figure 11:**
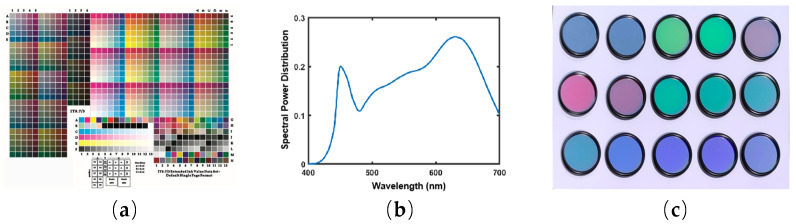
(**a**) IT8.7-3 CMYK target; (**b**) real spectral power distribution of the light source; (**c**) filters purchased in the laboratory.

This section involves the performing of real experiments in the dark room to further validate the proposed method. In this experiment, the IT8.7/3 color card is used as the data sample, which has 928 color blocks (Figure 11a). The response value of each color block are obtained using a Shot 5.0 multispectral camera with an ISO size of 50, an f-number-hole circle of F5.6, and an exposure time of 1/10 s. The real response values are extracted in the sRGB color space. The power distribution of the light source in the shooting environment is measured using the CS2000 spectroscopic radiometer, as shown in Figure 11b. The selection of the filter in the real experiment is still the same as in the simulation experiment, which is shown in Figure 11c.

#### 4.2.1. Experimental Environment

In order to obtain effective training sample color data and improve the accuracy of the data, a stable shooting environment must be determined before shooting.

**Figure 12:**
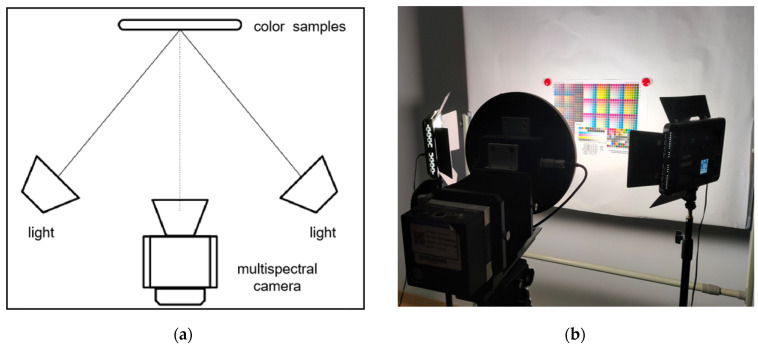
(**a**) Diagram of the shooting standard environment; (**b**) The real shooting environment.

First, in the process of shooting color pictures, the camera itself and the settings of the lighting and the surroundings are very critical. The stability of the lighting includes both time and space. If the amount of light radiation received by the target object surface changes over time, or the amount of light radiation received by the color sample at different spatial locations varies, then the camera response value signal generated by the color is bound to change as well. In addition, it is known from the optics of the camera that the light radiation energy received by the photoreceptor is strongest in the central part and decreases along the radius due to the optical effect of the convex lens inside the camera. Therefore, the color samples are placed in the center area of the camera’s field of view as much as possible during the shooting, and the training and test samples are placed in the center of the two light sources, ensuring that the light reached the samples from a 45° angle and placing the samples 1.5 m away from the camera lens, as shown in Figure 12a. From the time the light sources are turned on, they are warmed up for 30 min, and the light intensity of the light sources are allowed to stabilize before shooting. The above operation ensures the lighting stability from both space and time, and the real experimental scenario is shown in Figure 12b.

#### 4.2.2. Linear Calibration of Camera Response Values Correction

All of the experimental data in Section 4.1 are simulated experiments conducted under ideal conditions, thus ignoring noise, and assuming that a multispectral acquisition system has a perfect linear response model, which naturally produces smaller errors than the actual experiment.

**Figure 13:**
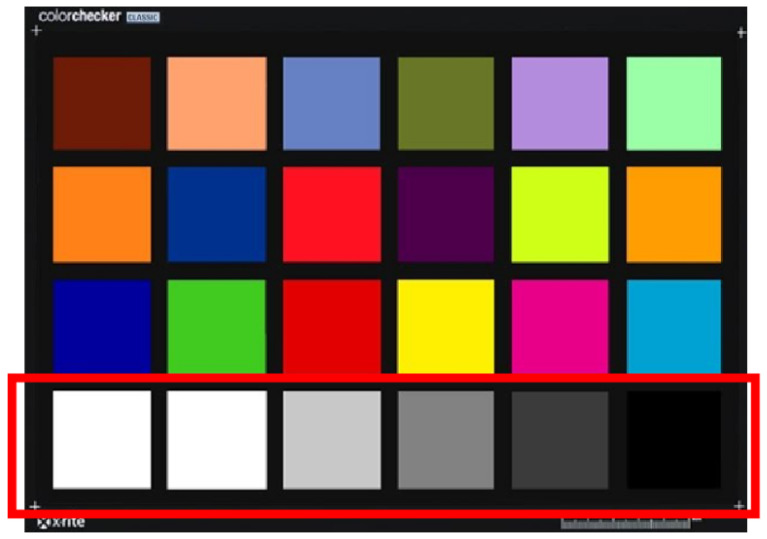
Color blocks 19 to 24 of the 24 color checker.

The response signal of the camera is obtained by the interaction of the light radiation energy incident on the sensor and the spectral response function of the camera, which is a set of linear data. However, in practice, the signal from the CCD or CMOS optical conversion of the multispectral camera will undergo a series of transmissions and compressions before it is outputted to the display device, at which time the response signal has become nonlinear data. If the camera signal acquired from the image is converted back to a linear response value [39], i.e., the digital signal of the camera is linearly corrected, then the spectrum recovered from the digital signal is as accurate as possible with the spectrum measured with a spectrometer, and an accurate spectral recovery effect is achieved. In this experiment, color blocks 19 to 24 of the 24 color card (Figure 13) are used as a grayscale, and Li’s [40] method is used to linearly correct the response values of the multispectral camera.

The photoelectric signal of the camera is linearly related to the optical radiation energy received by the CCD or CMOS, and the camera response value is also linearly related to the photoelectric signal of the camera. Therefore, this experiment achieves linear correction by establishing the conversion relationship between the camera response value and the CCD or CMOS optical radiation energy.

The light radiation energy T is obtained by the product of the spectral power distribution of the light source *E*(*λ*) and the reflectance of the color spectrum of the object surface *R*(*λ*) (see Formula (22)).

(22) $$T_{i}=\int E(\lambda )R(\lambda )d\lambda$$

**Figure 14:**
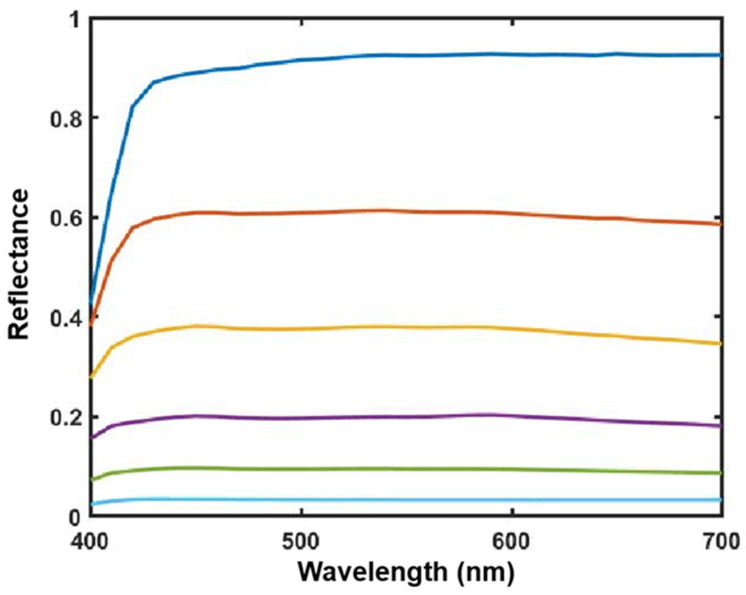
Spectral reflectance curves of gray samples.

Figure 14 shows the spectral reflectance curves of each gray sample, from which it can be seen that the reflectance curves of each color block remain basically horizontal, and the reflectance coefficients of each gray color block are similar. The six different color lines in Figure 14 represent the reflectance coefficients of the six gray color blocks.This indicates that the gradients of the gray color block we selected are uniform and reasonable, which can ensure the accuracy of the linear calibration data.

Before establishing the linear conversion formula between the light radiation energy and the camera response value, there is one more important operation, which is the normalization of the acquired data. The processed data will eventually be used to fit the linear formula. Table 4 shows an example of the normalization process for the experimental data of the third filter among the 15 filters to be selected.

T represents the light radiation energy distribution derived from Formula (21), and t represents the light radiation energy distribution after normalization with the maximum value of T as a reference. *P* represents the camera response value obtained by shooting, and *p* represents the response value of the brightest color sample (white) selected as a standard to normalize the response signal of the grayscale color and obtain the normalized response value.

In this experiment, the least squares method of curve fitting is used to obtain the linear conversion formula for each channel of the camera to achieve the linear correction of the digital signal. The linear conversion formula is established between the light radiation energy t and the camera response value, which can be realized by Formula (23).

(23) t=f(x)⋅p,

where *f*(*x*) represents the linear transformation formula, and the least squares implementation is implemented using the Matlab package. The final linear transformation formula selected was obtained by a Matlab calculation.

#### 4.2.3. Experimental Result

The linearly corrected camera response values are substituted into the multispectral imaging model (1), and subsequent calculations and selections are then performed as in the simulated experimental process to obtain the final experimental results.

The real experimental results are shown in Table 5. The results showed that the proposed method was consistent with the simulation experiments in spectral recovery and color difference, and both outperformed the existing methods, proving that the actual experiments achieved better results, and that the method proposed in this study can be applied to real scenarios.

**Figure 15:**
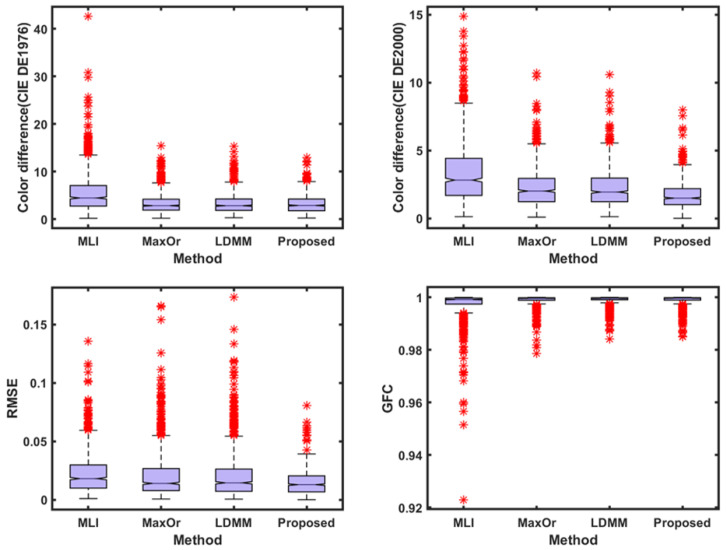
A box plot of each parameter index of the IT8.7/3 dataset under real experimental conditions.

**Figure 16:**
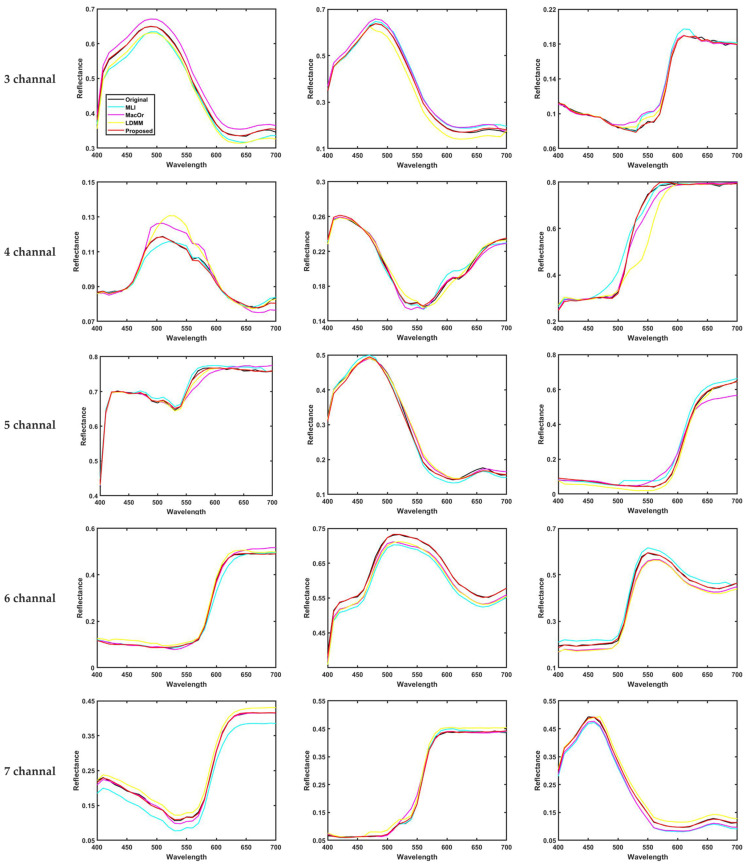
Spectral reflectance recovery results from our proposed and existing methods with three randomly selected samples.

Figure 15 shows the box plot distribution of the reference metrics for the present method compared with the existing methods, and Figure 16 shows the recovered spectral reflectance of three randomly selected detection samples, proving that the recovered spectral reflectance is more accurate and has a lower color error than the existing methods.

In summary, the high similarity between the simulated and actual experimental results confirms the superiority of this method.

## 5. Discussion and Conclusions

This study proposes a filter selection method that combines weighted area selection and custom error score ranking. The filter that best matches the human visual system is selected as the initial filter by weighting the filters using the LMS cone response function in combination with the area reducing rate selecting. The initial filter is combined with the remaining filters one by one, and the spectral recovery error and chromaticity error are calculated and then multiplied to select the filter combination with the smallest custom recovery error. In the experiment, we used four color samples and five channel types to verify the performance of the proposed method. The results show that using seven channels and choosing Munsell color samples for the experiments gives the best results with a mean root mean square error of 0.0031 and a mean color difference of 0.1566.

After validation by simulation and real experiments, the results show that the proposed method is better than other existing methods. By changing the data samples and the shooting environment, the method still outperforms other methods, showing good validity and robustness. This work will be useful for optimizing the spectral sensitivity of multispectral imaging sensors.

Due to the influence of the number of filters purchased by the laboratory, the number of filters to be screened in this experiment is 15, and the amount of data is relatively small. The feasibility of the proposed method was demonstrated by the validation of this experiment, and more filters will be purchased to continue to verify the generality of this method.

## Figures and Tables

**Table 1:** Results of different methods for recovery of the spectral reflectance of Munsell Matt chips.

Munsell Matt Chips									
Illuminant	Channel	Method	CIE DE1976		CIE DE2000		RMSE		GFC
			Max	Mean	Max	Mean	Max	Mean	Mean
CIE Illuminant A	3 Channel	LDMM	57.0162	5.1927	38.6427	3.8584	0.2281	0.0233	0.9932
		MLI	24.529	2.6168	14.0742	1.7299	0.1768	0.0153	0.9961
		MaxOr	57.0578	5.6398	38.6547	4.2504	0.2275	0.0254	0.9917
		Our	11.1701	1.3651	7.6661	0.9903	0.1135	0.0136	0.9975
	4 Channel	LDMM	33.0217	4.4186	27.2045	3.0929	0.1221	0.0156	0.997
		MLI	19.9484	1.4252	10.4861	0.9279	0.1262	0.0086	0.9987
		MaxOr	13.2427	1.5711	8.0956	1.0936	0.1222	0.0114	0.9982
		Our	7.1393	0.5538	5.1553	0.39	0.1029	0.0084	0.9988
	5 Channel	LDMM	13.5099	1.5452	7.3899	1.0844	0.0753	0.0082	0.9993
		MLI	20.2998	0.9775	10.7919	0.6506	0.0845	0.0054	0.9994
		MaxOr	13.1093	1.4526	7.4256	1.0421	0.1314	0.0107	0.9984
		Our	8.7672	0.5783	6.2174	0.4345	0.0456	0.0057	0.9996
	6 Channel	LDMM	4.3994	0.5009	2.7248	0.3787	0.0737	0.0046	0.9996
		MLI	19.6554	0.6257	10.4579	0.4594	0.0809	0.0045	0.9995
		MaxOr	11.0995	0.7065	7.1919	0.523	0.1222	0.0064	0.9992
		Our	3.461	0.2259	2.3218	0.1543	0.0238	0.0039	0.9998
	7 Channel	LDMM	1.7886	0.4236	1.3172	0.3571	0.0292	0.0037	0.9998
		MLI	19.7661	0.7179	22.5273	0.5512	0.0397	0.0039	0.9997
		MaxOr	6.6673	0.6496	4.4312	0.4812	0.0393	0.0041	0.9997
		Our	1.3168	0.207	1.1546	0.1566	0.0235	0.0031	0.9999

**Table 2:** Results of different methods for restoring the spectral reflectance of Color Checker SG.

Color Checker SG									
Illuminant	Channel	Method	CIE DE1976		CIE DE2000		RMSE		GFC
			Max	Mean	Max	Mean	Max	Mean	Mean
CIE Illuminant A	3 Channel	LDMM	55.5548	10.0086	33.767	7.2119	0.2251	0.0451	0.9811
		MLI	39.7361	7.2702	19.551	4.3265	0.1211	0.031	0.9924
		MaxOr	63.8962	8.7004	39.3476	5.8597	0.2407	0.0425	0.9811
		Our	18.088	2.4002	8.4941	1.4732	0.118	0.0267	0.9929
	4 Channel	LDMM	36.1135	7.1297	20.2838	5.405	0.0955	0.0286	0.994
		MLI	34.7927	4.8944	11.4471	2.7745	0.0992	0.0222	0.9957
		MaxOr	17.6933	2.7797	8.8284	1.826	0.1149	0.0258	0.9942
		Our	5.9632	1.0257	3.6337	0.6766	0.0931	0.0201	0.9962
	5 Channel	LDMM	11.7749	2.9412	9.3622	2.1346	0.072	0.0188	0.9978
		MLI	17.6457	3.722	7.6177	2.3171	0.1382	0.0198	0.9967
		MaxOr	18.8567	3.4702	12.3189	2.5038	0.1591	0.0264	0.9931
		Our	4.3094	0.9334	2.5814	0.6017	0.049	0.015	0.9986
	6 Channel	LDMM	4.9499	1.1014	2.9187	0.8902	0.0463	0.0131	0.9987
		MLI	19.3011	1.9112	8.4144	1.3322	0.1577	0.0136	0.9974
		MaxOr	6.8946	1.4448	4.477	1.0773	0.0768	0.0151	0.9974
		Our	2.3641	0.4807	1.2719	0.3264	0.0443	0.0129	0.9989
	7 Channel	LDMM	2.943	0.5482	1.1898	0.3772	0.0339	0.0096	0.9994
		MLI	9.7884	1.3752	5.7661	0.9455	0.0429	0.0084	0.9992
		MaxOr	11.8516	1.3307	4.8524	1.0435	0.0408	0.0103	0.9992
		Our	2.5998	0.3604	0.9242	0.2355	0.0256	0.0071	0.9995

**Table 3:** Results of different methods for restoring the spectral reflectance of the Vrhel spectral dataset.

Vrhel Spectral Dataset									
Illuminant	Channel	Method	CIE DE1976		CIE DE2000		RMSE		GFC
			Max	Mean	Max	Mean	Max	Mean	Mean
CIE Illuminant A	3 Channel	LDMM	55.2149	13.4954	35.9918	9.631	0.209	0.0576	0.9659
		MLI	33.342	9.4129	17.5367	5.031	0.1967	0.0351	0.9841
		MaxOr	56.4556	11.6292	36.4524	8.2406	0.1992	0.0521	0.9709
		Our	18.3209	2.6738	8.7486	1.6532	0.1818	0.0319	0.9862
	4 Channel	LDMM	70.5002	10.0769	28.8346	7.1759	0.2316	0.0362	0.9853
		MLI	29.8968	6.5727	13.2888	3.2126	0.1687	0.0286	0.9804
		MaxOr	25.6143	3.7521	15.2366	2.4752	0.1792	0.0328	0.987
		Our	18.2029	1.4805	8.213	0.91	0.167	0.0276	0.9894
	5 Channel	LDMM	27.6785	5.5163	14.3806	4.0168	0.1203	0.0254	0.9921
		MLI	32.3953	5.2023	13.698	2.6936	0.1529	0.0252	0.9904
		MaxOr	23.524	4.2321	14.2079	2.9	0.2085	0.0398	0.9763
		Our	11.4684	1.5269	6.2996	0.9421	0.1173	0.0177	0.9955
	6 Channel	LDMM	8.7184	1.3675	5.8822	0.9994	0.1062	0.0193	0.9945
		MLI	16.9549	2.8241	10.3674	1.7117	0.1538	0.0204	0.9917
		MaxOr	21.1351	2.3253	12.4645	1.4938	0.1706	0.0226	0.9893
		Our	7.9256	0.8298	3.2125	0.4499	0.102	0.0149	0.995
	7 Channel	LDMM	7.2469	0.7687	2.6931	0.4839	0.0871	0.016	0.9957
		MLI	39.0504	2.1657	18.9024	1.4431	0.1144	0.0122	0.9917
		MaxOr	10.8561	1.6483	7.202	1.1442	0.0884	0.0125	0.9945
		Our	6.981	0.6664	1.6818	0.3561	0.0821	0.0113	0.9964

**Table 4:** Light radiation energy T and camera response values for each gray sample.

NO.	T	t	*P*	*p*
1	1.80	1	97.73	1
2	1.19	0.67	75.13	0.77
3	0.73	0.41	54.09	0.55
4	0.39	0.22	33.42	0.34
5	0.19	0.10	21.22	0.22
6	0.07	0.04	6.69	0.07

**Table 5:** Results of different methods to recover spectral reflectance using IT8.7/3 samples.

IT8.7/3									
Illuminant	Channel	Method	CIE DE1976		CIE DE2000		RMSE		GFC
			Max	Mean	Max	Mean	Max	Mean	Mean
Real Illuminant	3 Channel	LDMM	55.1072	12.1706	36.3163	9.0247	0.2433	0.0462	0.9833
		MLI	72.3144	12.0688	33.6507	7.2681	0.1371	0.0375	0.9852
		MaxOr	36.8347	7.7783	25.5001	5.67	0.2001	0.0339	0.9921
		Our	14.5346	3.5781	9.8733	2.5609	0.1365	0.0237	0.9985
	4 Channel	LDMM	60.3669	8.6533	40.3494	6.4562	0.1732	0.0332	0.9896
		MLI	51.0632	10.5935	31.5347	6.2469	0.1404	0.0328	0.9883
		MaxOr	15.4071	3.4042	10.873	2.3232	0.1393	0.0229	0.9989
		Our	11.7962	2.9112	7.1387	2.0037	0.1382	0.0199	0.9992
	5 Channel	LDMM	15.352	3.2919	10.559	2.3487	0.1731	0.0216	0.9981
		MLI	42.5753	5.4712	14.8481	3.36	0.1357	0.0227	0.9974
		MaxOr	15.3741	3.3431	10.711	2.3564	0.1658	0.0218	0.9979
		Our	12.9502	3.2026	10.081	2.3046	0.1305	0.0215	0.9988
	6 Channel	LDMM	11.5558	2.6609	7.8024	1.8385	0.1456	0.0177	0.9991
		MLI	10.8503	2.8403	8.5263	1.9531	0.1164	0.0187	0.9991
		MaxOr	13.0843	2.7746	6.2731	1.8932	0.1239	0.0184	0.9992
		Our	10.4058	2.6028	7.6026	1.7872	0.1104	0.0163	0.9993
	7 Channel	LDMM	11.175	2.8872	8.0676	1.8862	0.1416	0.0174	0.9991
		MLI	11.7824	2.7372	7.3687	1.888	0.1133	0.0179	0.9992
		MaxOr	11.9103	2.6994	7.061	1.8502	0.1423	0.0173	0.999
		Our	10.91	2.6887	6.4923	1.8391	0.1107	0.0162	0.9992

## Data Availability

The data presented in this paper are not publicly available at this time but may be obtained from the authors upon reasonable request.
